# Demonstration of higher colour response with ambient refractive index in *Papilio blumei* as compared to *Morpho* *rhetenor*

**DOI:** 10.1038/srep05591

**Published:** 2014-07-07

**Authors:** Wanlin Wang, Wang Zhang, Xiaotian Fang, Yiqiao Huang, Qinglei Liu, Jiajun Gu, Di Zhang

**Affiliations:** 1 State Key Laboratory of Metal Matrix Composites Shanghai Jiao Tong University 800 Dongchuan Road, Shanghai, P. R. China

## Abstract

Multilayer structures are known to produce vivid iridescent colouration in many butterflies. *Morpho* butterflies are well known for their high reflectance, which appears to remain high over a wide range of viewing angles. Thus these butterflies have served as the inspiration for sensing materials. Using microscopic images and videos, we visually demonstrate that the colour response with ambient refractive index of *Papilio blumei* is better than that of *Morpho rhetenor.* This result was also verified using measurements of the reflectance for different viewing angles. The finite-difference time-domain method was then used to simulate the microscopic pictures and reflections. Finally, the relationships between the structure, ambient refractive index, reflection and viewing angle are discussed in detail.

Nanofabrication has been successful in creating photonic structures for chemical and biological detection^1^,^2^,^3^,^4^. An advantage of these photonic structures over organic dyes is the elimination of photobleaching problems. The main limitation of existing nanofabricated photonic-sensing materials is their low response selectivity to different analytes. Thus, their selectivity is conventionally enhanced using chemically selective moieties or layers^1^,^5^. However there is only a limited commonality between known biological and engineering solutions to challenging problems^6^, which suggests that natural structure would be used to solve this problem. The elaborate nanostructures in butterflies have been studied for use as sensing materials owing to their special optical effects^7^,^8^,^9^,^10^,^11^,^12^.

Multilayer structures are known to produce vivid iridescent colouration in certain butterflies^13^,^14^,^15^. These structures exhibit several variations based on two central design themes^16^. The first, called class I or the *Morpho* type^14^, is comprised of layering within discrete ridged structures on the surface of the scales that cover the wing. The second, referred to as class II or the *Urania* type^14^, is comprised of continuous multilayering within the body of the iridescent scales.

*Morpho* butterflies are rather unique in their high reflectance and in that their reflectance appears to remain high over a wide range of viewing angles, contrary to what is often observed from other natural examples of structural colours. This iridescence has been studied both in measurement and modelling techniques^17^,^18^. Thus the tree-like structure of *Morpho* butterflies has been investigated for use in sensing materials^7^,^9^,^11^,^12^.

The swallowtail family of butterflies (subfamily Lepidoptera of the family Papilionidae) is particularly renowned for its striking colour. Among the swallowtail butterflies, the brilliant green wings are found. The wing scales of of these butterflies consist of regular concave multilayer stack that are made from alternating chitin and chitin-air layers. One interesting phenomenon produced by this structure is that when placing the wing scales between crossed polarizers, light reflected off the centres of the cavities is suppressed, whereas retro-reflected light from four segments of the cavity edges is detected^19^,^20^. The graceful structure of these butterfly wings has been investigated^21^,^22^,^23^,^24^ and replicated by several researchers^25^,^26^,^27^,^28^,^29^.

The optical properties of *Morpho* butterflies in ethanol have been investigated by several researchers^7^,^30^,^31^. The results of these studies showed that, upon interaction with different ambient materials, the *Morpho* butterflies produce remarkably diverse differential reflectance spectra, achieving a highly selective response to different ambient refractive indices. In this paper, we will demonstrate that the response of *Papilio blumei* (*P.B.*) is better than that of *Morpho rhetenor* (*M.R.*) both in reflective intensity and viewing angle range.

The concave structure and the tree-like structure of *P.B.* (class II) and *M.R.* (class I) respectively, were chosen for this study. We demonstrated the obvious changes in colour with volatilization of butterfly scales in ethanol. The reflective properties of these structures with different viewing angles were also studied. While both structure types were very sensitive to ethanol, the most interesting result was that the sensitivity of *P.B.* was very different than that of *M.R.*. This difference in colour response with ambient refractive index (RI) arose as a result of their different structures. We then simulated the optical responses of the volatilization of two different structures using the finite-difference time-domain (FDTD) method. The simulation results reproduced the experimentally observed reflections, including the reflections observed for different viewing angles. We found that the colour response with ambient RI of the concave structure is better than that of the tree-like structure, and thus we performed a number of additional simulations to investigate the reason for this difference. Finally, the key parameters of the concave structure were identified to assist in the design of the sensing materials.

## Results

### Experimental data

**Figure 1:**
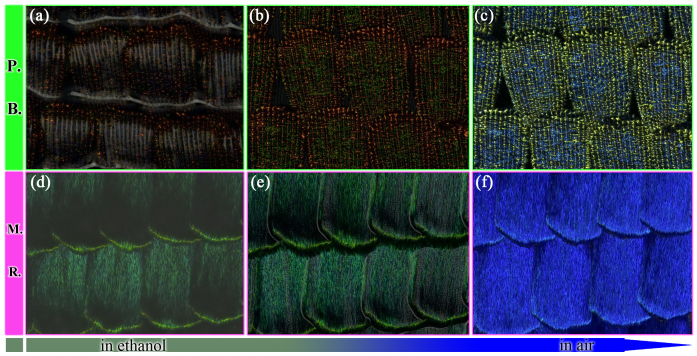
The microcosmic view of scales with the volatilization (a)(b)(c) with the volatilization of *P.B.*; (d)(e)(f) with the volatilization of *M.R.* The videos of the processes of volatilization are shown in Supplementary data. Normal incidence is set.

Figure 1 shows the microscopic view of the scales with the volatilization of *P.B.* and *M.R.* The colour of *P.B.* changed greatly, from dark yellow to green, while the colour of *M.R.* changed from green to blue. The whole process of volatilization is shown in the video files in the supplementary data. We could clearly see that the colour responses of both butterflies were very vigorous, with the colour response of *P.B.* being more vigorous than that of *M.R.*. It is generally understood that these colour responses are result of their structure. Thus detailed simulations were performed to investigate the difference in the colour response of the two types of structure.

**Figure 2:**
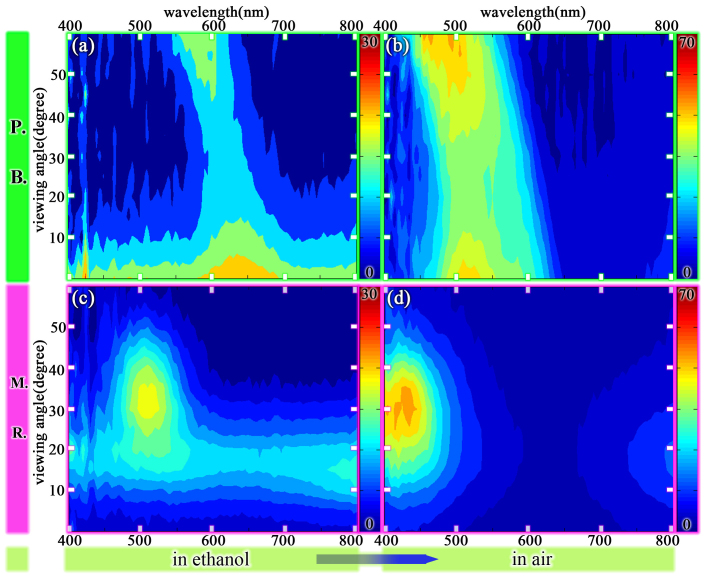
The contour plot of reflectance spectra of *P.B*. and *M.R.* versus wavelengths and viewing angles in ethanol and in air. (a)(b) *P.B.* in ethanol and in air; (c)(d) *M.R.* in ethanol and in air.

The colours of *P.B.* and *M.R.* are dependent on viewing angle. Thus the viewing angle dependence of both colour responses were measured. As shown in Figure 2(c) and (d), the viewing angle range of *M.R.* in air and in ethanol shifted from 27° to 24°, which is a fairly small shift. A similar result for *M.R.* was derived from experimental data (using only 488 nm incident light)^31^. In contrast, the viewing angle range of *P.B.* in air and in ethanol is shifted from 60° to 5° as shown in Figure 2(a)(b). The shift is much larger than that of *M.R.*. Also we can see that the viewing angle range of *P.B.* is larger than that of *M.R.* in air, while the viewing angle range of *M.R.* is bigger than that of *P.B.* in ethanol. Thus the colour response of *P.B.* is more vigorous than that of *M.R.*. The reasons for this phenomenon will be discussed subsequently.

### The structure of *P.B.* and *M.R.*

**Figure 3:**
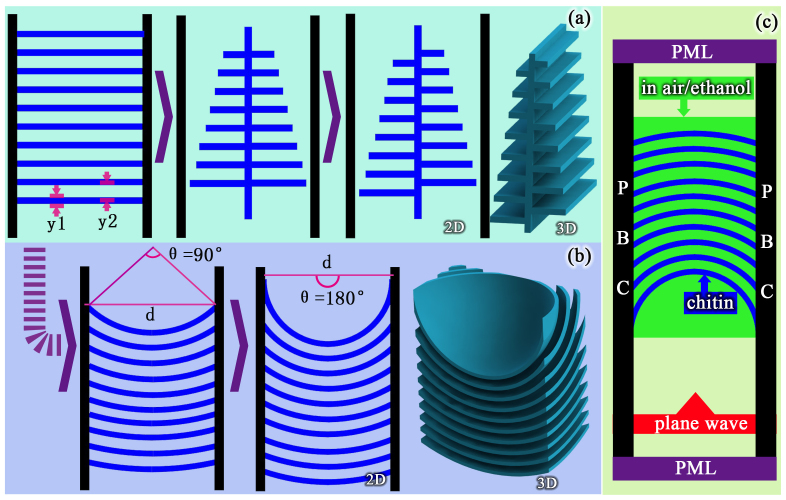
The models of *P.B.* and *M.R.* and the boundary conditions. (a) the 2D and 3D models of tree-like structure evolved from multilayer (b) the 2D and 3D models of concave structure evolved from multilayer. The parameters y1, y2, d and θ is shown. The 3-D model is shown in right. (c) the boundary conditions in vertical direction is absorbing (perfectly matched layer, PML) and in horizontal direction is periodic (periodic boundary condition, PBC). The blue colour is chitin and the green colour is air or ethanol.

The models shown in Figure 3 were constructed according to their electron microscope images^22^,^31^. As shown in Figure 3(a) and (b), both the tree-like and the concave structures can be evolved from a multilayer structures. However, the parameters y1 and y2 (illustrated in Figure 3(a)) were defined to be 60 nm and 140 nm in the tree-like structure, while they were defined to be 70 nm and 160 nm in the concave structure. The variation in these parameters is the reason for the different colours of these two butterflies. The field angle (θ) and period (d) of concave structure will be discussed in detail subsequently. The boundary conditions of the simulation are shown in Figure 3(c); these conditions were absorbing (perfectly matched layer, PML) in the vertical direction and periodic (periodic boundary condition, PBC) in the horizontal direction. We assumed that the refractive index of chitin was 1.56 + i0.06, in accordance with the results reported in the literature^31^,^32^,^33^. The refractive index of ethanol was set as to 1.361 in the volatilization simulation.

### Simulations performed to reproduce the observational data

**Figure 4:**
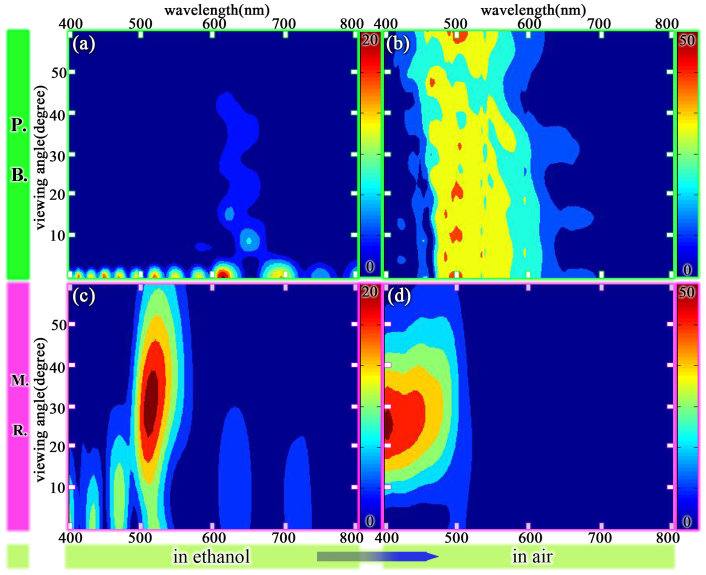
The simulation of reflectance spectrum versus viewing angle (a)(b) *P.B.* in ethanol and in air (c)(d) *M.R.* in ethanol and in air.

In order to study the colour response of the volatilization of both structures, we defined a liquid level x1 range from 0 nm to 5000 nm as shown in Figure 4(a). Next, we simulated the process of volatilization using the FDTD method. The reflectance spectra versus wavelength and x1 are shown as contour plots in Figure S1 (b) and (c). The colour is shown in colour-maps which are calculated from their reflectance spectra. And the corresponding method is given below. The results demonstrate that the reflectance spectra changed sharply with the volatilization of ethanol. Specifically, the peak intensities of the reflectance increased and the peak sites of the reflectance shifted greatly with the volatilization of ethanol. And also the peak sites of their reflectance shift greatly with volatilization. It can be observed that the colour changed abruptly with volatilization in both structures, as shown by the colour-maps in Figure S1 (b) and (c). This result reproduces the phenomenon observed in video files in Supplementary data. Both the increase in the peak intensity of the reflectance and the shift in the peak reflectance site are larger for the concave structure than for the tree-like structure. Thus the colour response with ambient RI of the concave structure is larger than that of tree-like structure, confirming the results derived from the experimental data.

The angular dependence of the colour response of both structures discussed above is shown in Figure 4. These results confirm that there is a significant colour response of the concave structure with different ambient RI, and also confirm the existence of a less evident colour response of the tree-like structure with different ambient RI. Furthermore, the viewing angle range of the concave structure is larger than that of the tree-like structure. In summary, these results demonstrate that the colour response of the concave structure is more vigorous than that of the tree-like structure. Thus, in the following sections we discuss the model of the concave structure in order to determine the cause of this heightened colour response.

### Sensitivity of the reflection intensity to ambient RI

**Figure 5:**
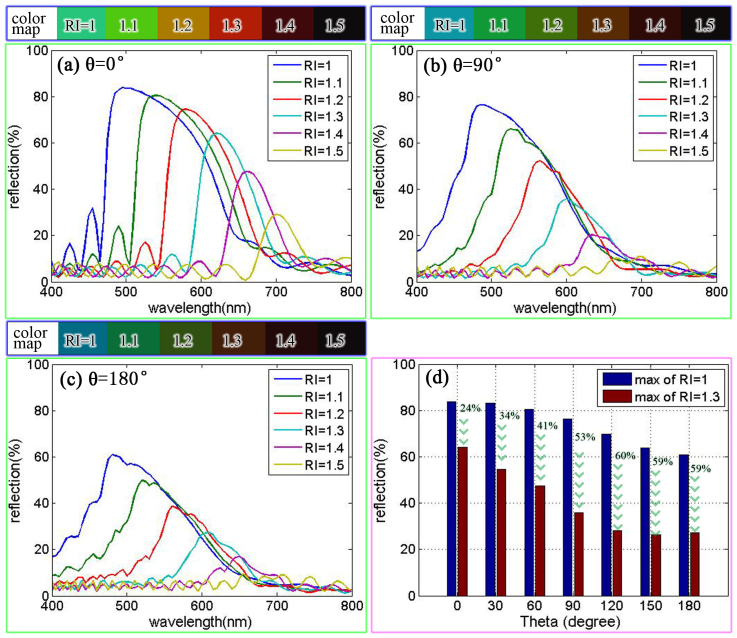
Reflectance spectrum with different field angle (θ) of concave structure (a) line plot with θ = 0° (b) line plot with θ = 90° (c) line plot with θ = 180° (d) bar graph of reflectance with different field angle, the drop rate is shown respectively. The colour-maps above (a)(b)(c) are calculated form corresponding reflectance spectrum.

Reflectance spectra for different field angles (θ) of the concave structure are shown in Figure 5. To understand the relationship between the colour response, field angle, and the ambient RI, following observational characteristics. (1) The reflectance decreased with increasing of RI. (2) The drop rate of the reflectance (between RI = 1 and RI = 1.3) decreased as field angle (θ) increased. (3) The highest drop rate (between RI = 1 and RI = 1.3) was located from 90° to 120°. Thus the colour response as a function of the ambient RI is related to the field angle of the concave structure. The colour-maps which are calculated from reflectance spectra shows the colour response more visualized. The reflectance spectra in air and in ethanol for the structures evolving from a multilayer to a tree-like structure are shown in Figure S2. This result shows that the tree-like structure did not enhance the colour response. This confirms that the colour response with different ambient RI is more vigorous for *P.B.* than for *M.R.*.

**Figure 6:**
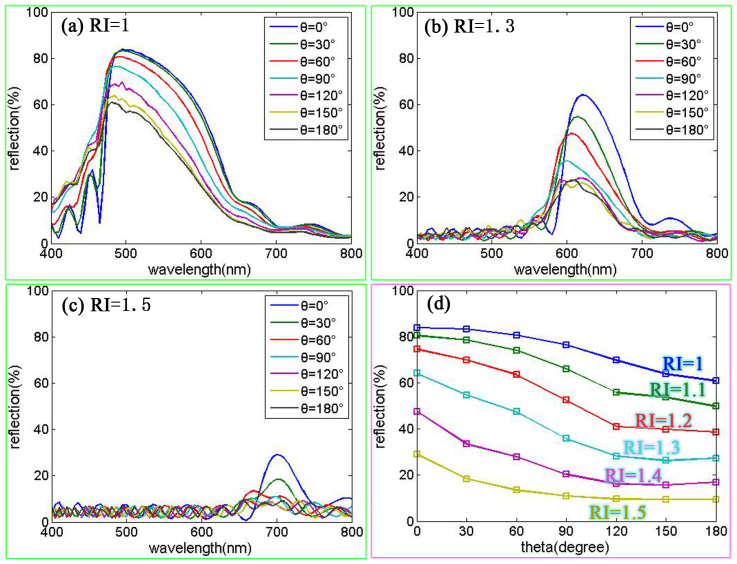
Reflectance spectrum with different ambient RI (a) line plot versus different wavelength with RI = 1 (b) line plot versus different wavelength with RI = 1.3 (c) line plot versus different wavelength with RI = 1.5 (d) line plot versus different θ with different RI.

In order to display the relationship between the RI and the field angle more clearly, reflectance spectra for different ambient RI are shown in Figure 6. We can derive conclusions from the following characteristics: (1) the reflectance decreased with increasing field angle, and (2) this decrease became very small when the field angle increased to a value which is determined by the RI. We see that the reflectance spectrum depends upon the RI and field angle; thus, the colour response is also related to the RI and the field angle. As such, to achieve good colour response, the structure should be carefully designed with the proper ambient RI and the proper field angle.

### viewing angle sensitivity of reflection to ambient RI

**Figure 7:**
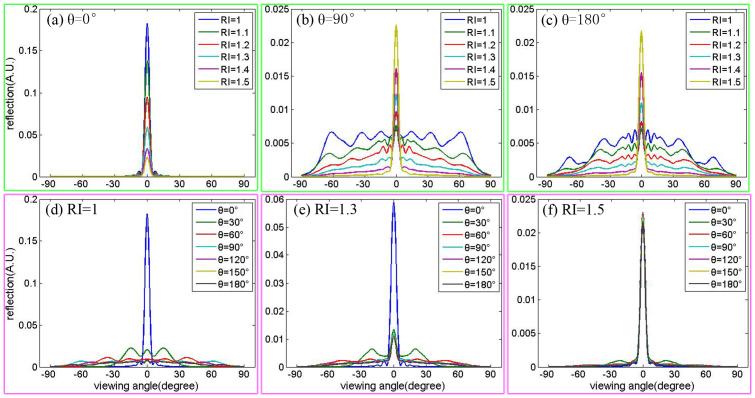
Line plot of reflectance versus viewing angle with different ambient RI. (a)(b)(c) θ = 0°, 90° and 180° of concave structure; (d)(e)(f) RI = 1, RI = 1.3 and RI = 1.5 of concave structure.

The viewing angle dependencies of multilayer structure to concave structure were measured and simulated as described above. Here we examine this issue in further detail, as shown in Figure 7. For a variety of ambient RI values, the viewing angle range of the multilayer structure (θ = 0°) was very small, as shown in Figure 7(a). High ambient RI values improved the intensity of the reflectance, while, this improvement in the reflectance intensity decreased with higher field angles. For a range of ambient RI values, the improvement in the reflectivity at θ = 90° is smaller than that at θ = 0°, and there was only a little difference between θ = 90° and θ = 180°. The viewing angle range enlarged with increasing field angle as shown in Figure 7(d); thus the viewing angle range of a concave structure is wider than that of a multilayer structure(θ = 0°). For larger RI values the effluence of the field angle on the viewing angle range became smaller. As shown in Figure 7(f), for RI = 1.5 the reflectance was almost the same for all field angles. For concave structures, the viewing angle range is affected by the ambient RI and the field angle; thus, this factors should be taken into account carefully when we design such structures.

## Discussion

**Figure 8:**
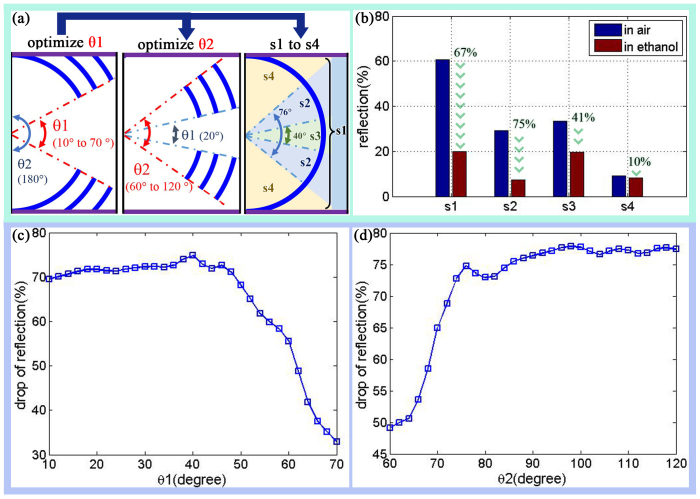
Optimization of concave structure with field angle (θ). (a) sketch map of optimization, the full concave structure named as s1, θ1 and θ2 cut apart s1 into three parts s2, s3 and s4. θ1 scan from 10° to 70°, θ2 scan from 60° to 120°. (b) bar graph of reflectance of s1, s2, s3 and s4, the drop rate (between in ethanol and in air) is shown correspondingly. (c) line plot of drop rate of reflection with scan θ1 from 10° to 70°. (d) line plot of drop rate of reflection with scan θ1 from 60° to 120°.

The concave structure has a vigorous colour response for different ambient RI as discussed above. This colour response is relative to the field angle. As shown in Figure 8(a), we optimized the concave structure (s1) by cutting it into three parts: s2, s3 and s4. The field angles θ1 and θ2 were then scanned from 10° to 70° and 60° to 120°, respectively, to search for the highest drop rate of the reflectance between ethanol and air, as shown in Figure 8(c) and (d). The influence of θ1 and θ2 on the drop rate of the reflectance is clear. The turning points for θ1 and θ2 are 40° and 76°. Thus structures s1, s2, s3 and s4 are defined from 0° to 180°, 40° to 76°, 0° to 40° and 76° to 180°, respectively. The bar graph in Figure 8(b) shows the reflectance of s1, s2, s3 and s4. The drop rate of s2 (75%) is higher than that of s3 (41%) and s1 (67%). The s2 is the key part for producing vigorous colour response of the concave structure. Note that the drop rate of s4 is very small (10%) between ethanol and air.

**Figure 9:**
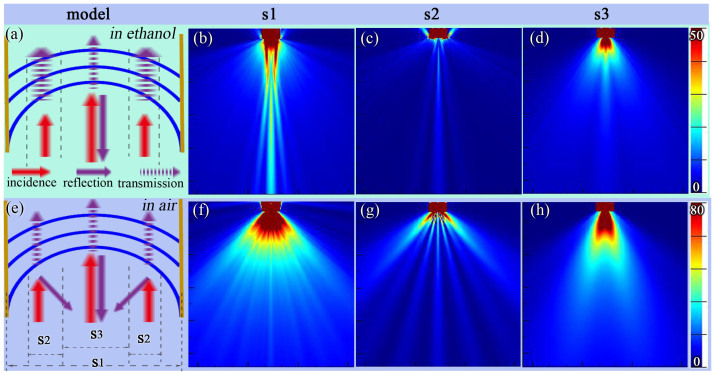
Electric field map with s1, s2 and s3 (a)(e) the model of concave structure in ethanol and in air (b)(c)(d) electric field map in ethanol with s1, s2 and s3 (f)(g)(h) electric field map in ethanol with s1, s2 and s3. Three different arrows are incidence, reflection and transmission light in (a)(e).

The electric field map is presented in Figure 9 to verify the conclusions derived for s1, s2 and s3. The viewing angle range is shown in each electric field map. The electric field map for s4 is pictured here because of its small drop rate and low reflectance. Figure 9(b) and (f) demonstrate that the viewing angle range in air is wider than that in ethanol. The viewing angle range in air of s1 is wide, encompassing the full range from the middle to two sides, while the viewing angle ranges of s2 and s3 in air are located primarily in the middle and at two sides, respectively. The reflection of s3 in ethanol changes little when compared with the reflection in air, thus, s3 is not the key component in the colour response. In contrast, the reflection in air of s2 has a wider viewing angle range and a higher intensity than in ethanol. Thus this structure is the reason for the colour response difference between air and ethanol.

## Method

### Experimental measurements

Digital microscopes achieve a larger depth-of-field than optical microscopes, allowing for accurate observation of a target with a highly uneven surface. In this study, the vivid observations and the corresponding video files of the colour response with different reflective indices were therefore obtained using a digital microscope. To make the results comparable to each other, the data were obtained under identical conditions, e.g. of brightness and depth-of-field.

Angle-dependent reflectance spectra of the butterflies were acquired with an angle-resolved reflectance measurement setup based on a pair of optical fibres that could be rotated independently around the same axis, one acting as the light source, the other as the light collector. A stabilized halogen light source were used for reflectance measurements. The butterflies were placed with their ridges parallel to the rotation axis, and the light reflectance spectra were measured as a function of the viewing angle. To measure the iridescence, the fibres of the light source were set at 10° from the centre, and the fibres of the light collector was rotated in opposite, equally spaced steps of 10° from 0° to 60°.

### From reflection to colour

In order to reproduce the colour response of the butterflies we converted reflection into colour maps. Let us suppose that the butterfly is illuminated by an illuminant characterized by its energy distribution D(λ). In this paper, we use the CIE (Commission Internationale de l'Éclairage) normalized illuminant D65, which closely matches that of the sky daylight^34^. Assuming that the butterfly has a reflectivity R(λ), we can compute the CIE XYZ tristimulus values which are one possible way to characterize the reflected colour. We then transform the XYZ components into RGB components, which are more convenient for the imaging process. The last step is the colour representation using MATLAB.

At this stage, we have replaced a complicated set of data (i.e. the spectrum R(λ)) by a colour point in a colour map. As illustrated in the above sections, this representation can be very useful for obtaining the dynamic reflection properties. A two-dimensional colour-map based on this representation has two free parameters (for example the dynamic reflection of the butterfly with the process of volatilization).

### Optical modelling

To theoretically validate the nature of the optical response, we performed optical and volatile modelling of the *M.R.* and *P.B.* nanostructure. In the optical model, we took into account two contributors to the optical changes: (i) the two different structure types (ii) a change in the refractive index induced by volatility. The scales of these two butterflies are formed largely from chitin, whose refractive index was assumed to be 1.56 + i0.06^31^,^32^,^33^. Most of the aforementioned geometrical data on *M.R.* were obtained from electron microscopy studies^31^,^35^, and the geometrical data of *P.B.* were obtained from electron microscopy studies^22^. The light scattering by the structure of the butterflies was simulated using the FDTD method. The reflection of the 3D concave structure, shown in Figure S3, was similar to the result for the 2D concave structure shown in Figure 6(a). In order to intensively study the parameters of the concave structure in a reasonable simulation time we studied this problem using the 2D structure. We defined the wavelength to be between 400 and 800 nm. The mesh size was chosen to obtain a good trade-off between the computer memory required and the simulation time, while ensuring convergence of the results. A convergence test was carefully performed.

## Author Contributions

W.L.W. and W.Z. contributed equally to this work and performed the experimental data. X.T.F., Y.Q.H. and Q.L.L. helped with data analysis and theoretical calculations. W.L.W., J.J.G. and D.Z. performed the FDTD simulations. W.L.W., W.Z. and D.Z. contributed to the writing of the manuscript.

## Supplementary Material

Supplementary Information the volatilization of PB

Supplementary Information the volatilization of MR

Supplementary Information Dataset
