# Supplementary material for: Demonstration of higher colour response with ambient refractive index in Papilio blumei as compared to Morpho rhetenor
Source: Sci Rep. 2014 Jul 7;4:5591. doi: 10.1038/srep05591 (PMC4083278; doi:10.1038/srep05591)
Supplement: Supplementary Information — Dataset [file srep05591-s3.doc]

Demonstration of higher colour response with ambient refractive index in *Papilio blumei* as compared to *Morpho* *rhetenor*

Wanlin Wang,^1^ Wang Zhang,^1,*^ Xiaotian Fang,^1^ Yiqiao Huang,^1^ Qinglei Liu,^1^ Jiajun Gu ^1^ and Di Zhang^1,*^

^1^ *State Key Laboratory of Metal Matrix Composites, Shanghai Jiao Tong University, Shanghai, 200240, China*


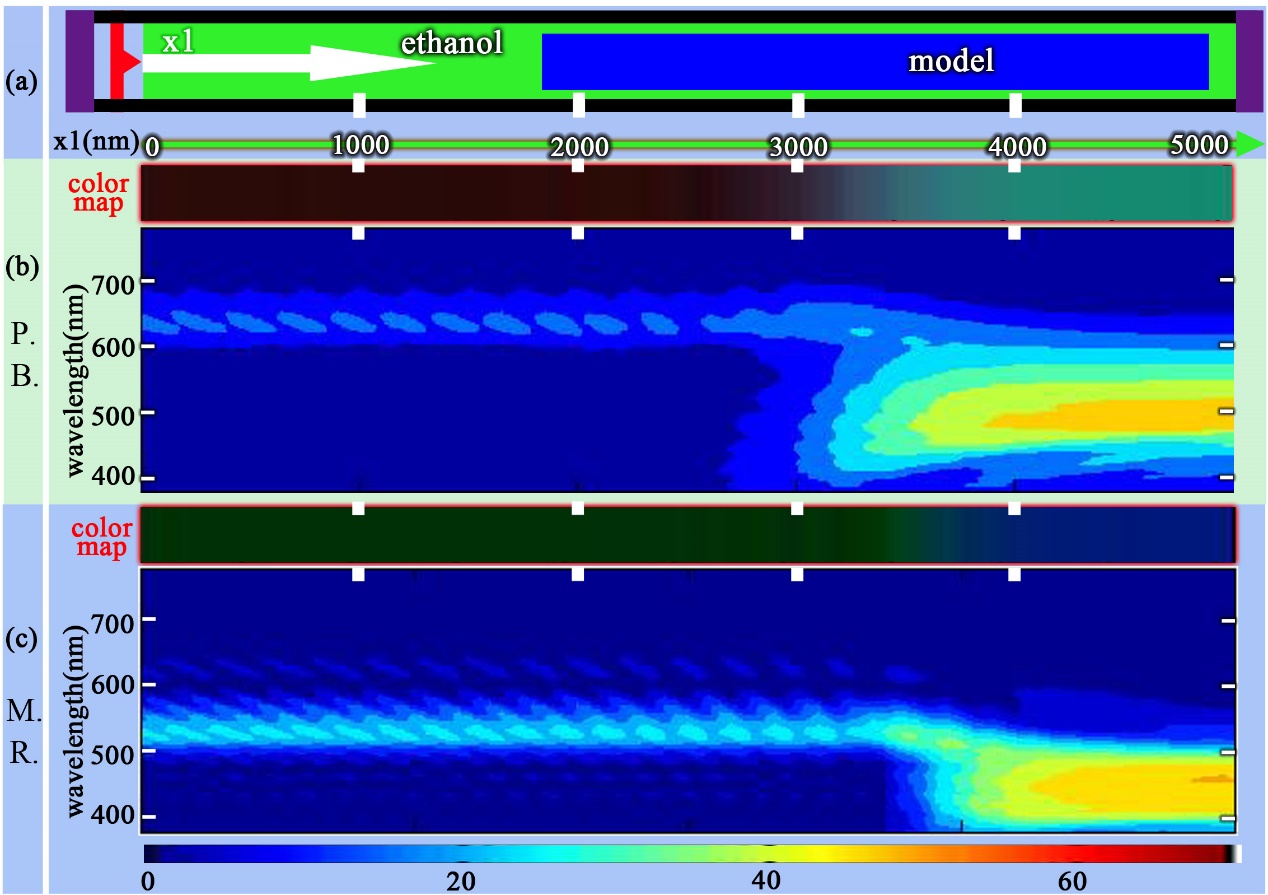


Figure S1 the simulation of the change of reflectance spectrum and corresponding colour-maps of the volatilization. (a) the model of the volatilization (b) contour plot of reflection and corresponding colour maps of P.B. (c) contour plot of reflection and corresponding colour map of M.R.. The colour-maps are calculated form their reflectance spectrum.


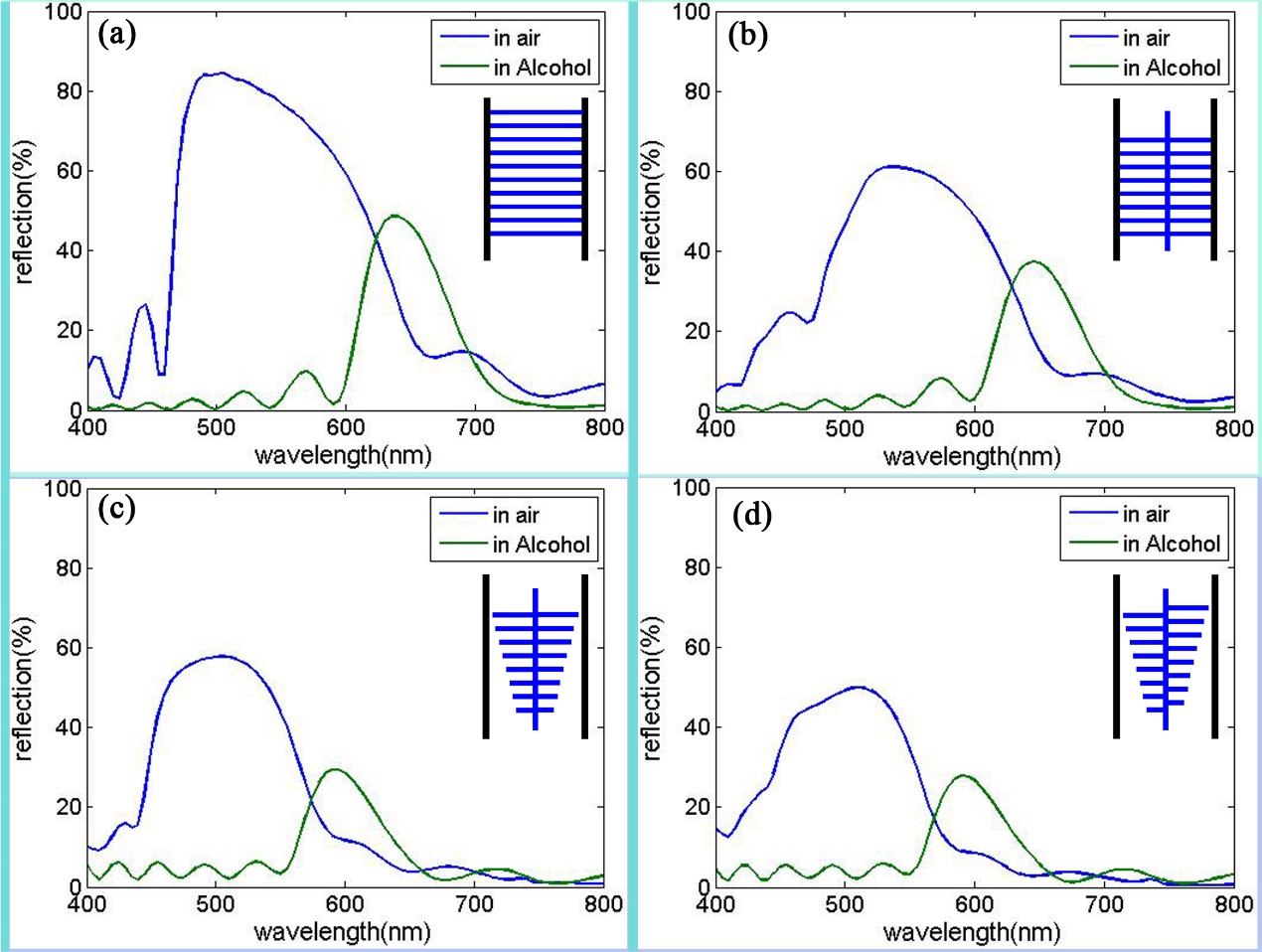


Figure S2 reflectance spectrum in air and in ethanol (a)(b)(c)(d)with the structure from multilayer to tree-like structure


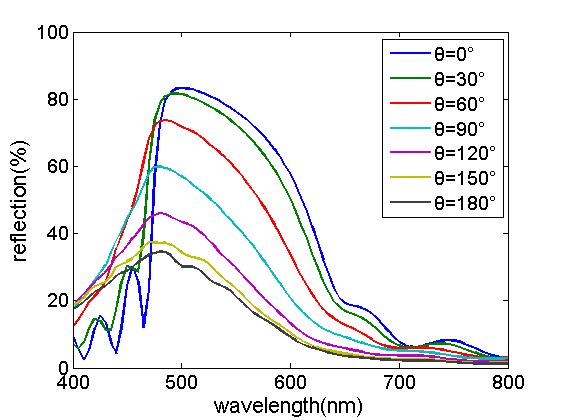


Figure S3 reflectance spectrum with different field angles (θ) of 3D concave structure with ambient RI=1.
